# Socio-economic and demographic impacts on the full awareness of the methods for controlling/preventing the spread of COVID-19 among social media users in some African countries at the onset of the pandemic

**DOI:** 10.1186/s13104-021-05736-z

**Published:** 2021-08-27

**Authors:** Idika E. Okorie, Emmanuel Afuecheta, Chinonso G. Alaebo, Saralees Nadarajah

**Affiliations:** 1 Department of Mathematics, Khalifa University, P.O. Box 127788, Abu Dhabi, UAE; 2 Department of Mathematics and Statistics, King Fahd University of Petroleum & Minerals, Dhahran, Saudi Arabia; 3 African Institute for Mathematical Sciences (AIMS), Mbour, Senegal; 4 Department of Mathematics, University of Manchester, Oxford Road, Manchester, M13 9PL UK

**Keywords:** COVID-19 pandemic, Social media, Socioeconomic and demographic characteristics, Regression analysis

## Abstract

**Objective:**

In Africa, most countries continue to battle COVID-19 with cases of newly infected still being recorded. In this note, we investigate how socioeconomic and demographic factors affected individuals awareness on the methods for controlling/preventing the spread of COVID-19 in some parts of Africa at the onset of the pandemic.

**Results:**

Based on regression modelling, we find that having full awareness does not depend on religious affiliation. Men, urban dwelling, holding bachelors or higher degrees, operating multiple social media accounts or being employed are associated with having full awareness of the recommended practices for the prevention and control of COVID-19 at the early stage of the pandemic. No occupation, business or older people are associated with not having full awareness.

**Supplementary Information:**

The online version contains supplementary material available at 10.1186/s13104-021-05736-z.

## Introduction

The COVID-19 pandemic sparked a global health crisis as well as human tragedy in most countries of the world. The pandemic commenced in Wuhan (China) and spread to America, Europe, and other parts of the world. Scientists worldwide scrambled to find a vaccine for this novel coronavirus. While this was ongoing globally, different countries adopted different mitigation measures recommended by the World Health Organization (WHO) to influence the course of the COVID-19 pandemic. Some of these measures included isolating people who contracted the virus, tracing and quarantining their contacts, social distancing, and practicing good hygiene (WHO 2019; [3]; UN News 2020; [5–9]). Remarkable among these measures is “social distancing” whose goal is to slow down the outbreak dynamics to avoid putting much pressure on an already strained health care system. By social distancing, the pandemic curve was shown to reduce drastically (Flatten the COVID-19 curve. https://www.r-bloggers.com/flatten-the-covid-19-curve/).

However, as noted by Miller et al. [14], the impact of this pandemic hit virtually all the countries with wildly different levels of severity perhaps due to variations in cultural norms, heath care system as well as alleviation endeavours. For example, social distancing and many public institutions’ closure present major challenges to engagement and society in many poor countries. This pandemic’s economic impact could skyrocket food insecurity in low income countries, which would likely increase vulnerability to disease (World Food Program WFP [22]).

Prior to COVID-19, severe food insecurity was most prevalent in Africa with more than 200 million hungry people (Food and Agriculture Organization- FAO [10]). The continent recorded her first case of the virus on the 14th of February 2020 in Egypt, followed by Algeria and Morocco. Since then, other countries have registered numerous cases and fatalities. As of 11 May 2020, there were more than 63,000 confirmed cases, 2290 deaths, and 21,837 recoveries, cutting across the entire continent with Lesotho the only country holding out [1]. South Africa is the most affected country in the region, with over 10,000 cases. However, these figures may not be a true reflection of the situation on the ground presently, given the large gap in testing rates between countries. Innovative measures are required to prevent the entire continent from becoming hot spots of COVID-19.

Therefore, at the early stage of the pandemic, to significantly and effectively slow down the spread of the virus in Africa, it was crucial that everyone observed the necessary precautionary measures. As such, scaling-up COVID-19 awareness and the methods for control/prevention by appropriate authorities were very important. However, mediating factors such as socioeconomic and demographic characteristics are inherent to the decision-making process of COVID-19 measures. Thus, the study these factors is important [3, 6, 7].

The purpose of this note is to investigate how socioeconomic and demographic factors affected the full awareness of the methods for controlling/preventing the spread of COVID-19 in some parts of Africa at the early stage of the pandemic. The findings in this note will help policymakers to: make informed, evidence-based decisions as the COVID-19 pandemic evolves from both practical and economic perspectives; understand some uncertainties associated with this virus (for example, factors that explain the regional differentials in population vulnerability); prepare for future pandemics. This note includes the materials and methods section; results, discussion and conclusions section; section on limitations.

## Main text

### Materials and methods

Let *Y* denote a Bernoulli random variable, we can refer *Y* as an outcome/response variable. Let *X* denote the independent/predictor variable, *X* could either be a numeric or a categorical variable. Then, the logistic regression model with a logit link (which is a natural model for this kind of response variable) can be specified as

$$\begin{aligned} {} & y_i = \pi ( x_i ) +\xi _i\sim {\text{Binomial}} ( 1, \pi ( x_i ) ) ,\\ & \log \left[ \frac{\pi ( x_i) }{1 - \pi ( x_i) }\right] = \beta _0+\beta _1x_{1, i}+\beta _2x_{2, i}+\cdots +\beta _px_{p, i}, \ i=1,2,\ldots ,n, \end{aligned}$$

where $\pi ( x_i ) = {\text{Pr}} \left( Y_i=1|X=x_i\right)$ is the conditional probability of the outcome $y_i$ given the predictor $x_i$, $\beta$s denote the model parameters, *p* denotes the number of predictors, *n* denotes the sample size, and $\xi$s represent the error terms. For detailed information on the logistic regression model, we refer to Hosmer et al. [12]. The fitting of the model was performed by the ‘glm’ function in the ‘stat’ package of $\textsf {R}$ ($\textsf {R}$ Core Team [17]).

**Fig. 1:**
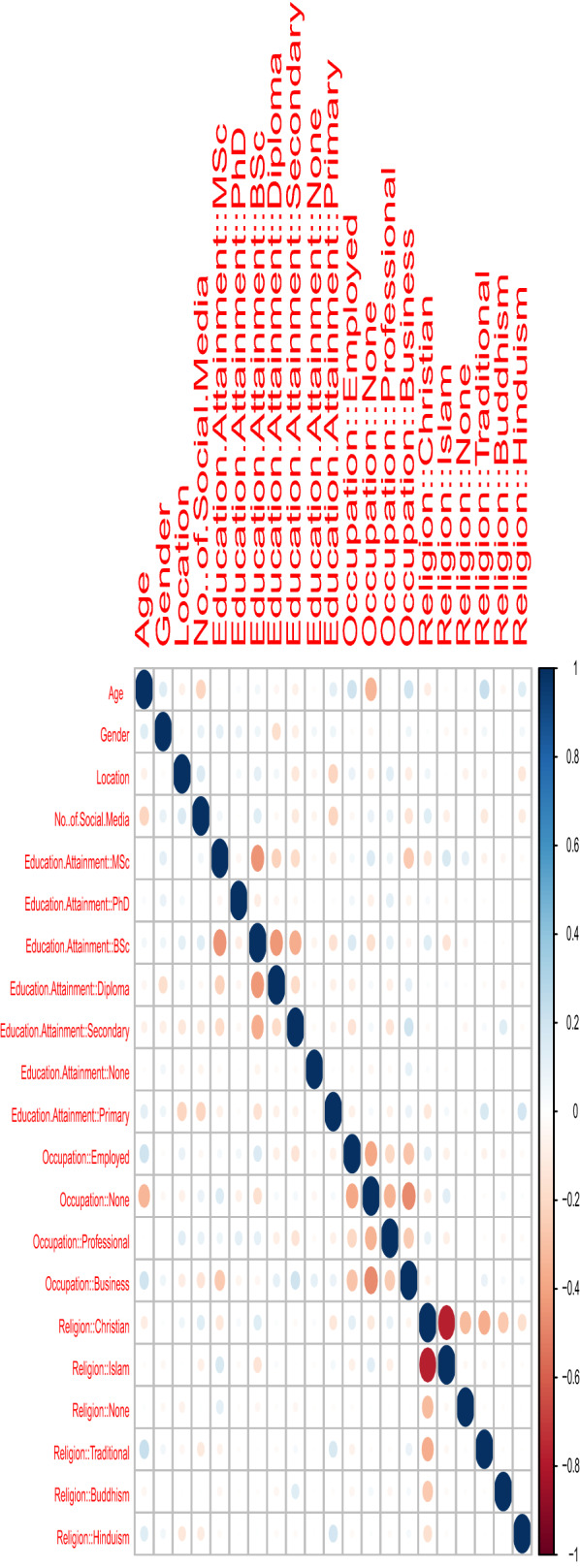
Correlation matrix for the predictors

We need data on *X* and *Y*. Africa is a large continent; so, there will be an enormous financial involvement in hiring ad-hoc staff, travelling from one African country to another, accommodation and additional constraints. To circumvent these limitations, we solicited social media users to complete questionnaires online on Facebook, WhatsApp, Twitter and Instagram. Only people domiciled in Africa at the time of the survey were eligible to participate. The minimum age of consent varies across Africa in the range 12–18 (https://en.wikipedia.org/wiki/Ages_of_consent_in_Africa); only data involving participants aged 18 or above were considered for analysis. At the end of the data collection exercise, we received 537 (*n*) duly completed questionnaires. The data collection took place between 07-04-2020 and 28-06-2020. To avoid the issue of missing data, we ensured that all questions in the questionnaire were made mandatory for the respondents to answer, so that only duly completed questionnaires can be submitted. Several mandatory questions take about 8 minutes to answer; so, we believe that only genuine and dedicated respondents can endure to complete the questionnaires, guaranteeing the validity and reliability of the responses. The African nations that were represented in the survey are (number of participant(s) in parenthesis): Benin Republic (1), Burkina Faso (2), Burundi (1), Cameroon (27), Congo (3), Gambia (1), Ghana (21), Kenya (7), Nigeria (436), Rwanda (2), Senegal (22), South Africa (3), Sudan (3), Togo (4), Uganda (2), and Zambia (2). The responses from the questionnaire are summarized in Additional file 1.

There are numerical and categorical responses from the questionnaire. We coded the binary categorical responses as follows: Awareness level (0 for no full awareness and 1 for full awareness); Gender (0 for female and 1 for male); Location (0 for rural and 1 for urban). We used the dummy variable encoding method for other categorical responses taking more than two levels (education attainment, occupation, and religion). We take Awareness as the outcome variable *Y* and remaining as predictor variables *X*.

Multicollinearity is a prominent problem in regression analysis, see Alin [2]. To build a model that is void of multicollinearity, only uncorrelated predictors should be included. For the selection of predictors, we use the correlation coefficient ($\rho$) matrix in Fig. 1; for any two predictors having a significant correlation, only one of them is included in the model. If $|\rho |<0.3$, we say that the correlation is negligible, see Mukaka [16]. From Fig. 1, we see that Christianity and Islam are negatively correlated (${\widehat{\rho }}=-0.775$); business is negatively correlated with no occupation (${\widehat{\rho }}=-0.478$); MSc degree, diploma certificate and secondary school are negatively correlated with BSc degree (${\widehat{\rho }}=-0.444$, ${\widehat{\rho }}=-0.433$ and ${\widehat{\rho }}=-0.367$, respectively); traditional worshipping and Christianity are negatively correlated (with ${\widehat{\rho }}=-0.361$); Christianity and no religion are negatively correlated (${\widehat{\rho }}=-0.318$). Thus, we do not include Christianity, business, diploma certificate, no religion and no occupation variables in the model. Finally, we use the variance inflation factor (VIF) to measure the strength of correlation between the selected predictor variables in the regression model. The outcome is listed in Table 1. All the VIF values in Table 1 are $<5$; thus, they do not raise any alarm against multicollinearity.

### Results, discussion and conclusions

The fitted model in Table 1 gave a residual deviance of 440.80 with 521 degrees of freedom and an associated $p-$value of $0.9953915(>0.10)$. This indicates no significant evidence against the fitted model. The McFadden pseudo R-squared value of the fitted model is 0.1205170, indicating a decent fit.

In the model building process, the logistic regression model with an intercept was initially fitted to the data; but, some of the predictors that were statistically significant in the refined model without intercept in Table 1 turned out to be insignificant. This goes to show that some of the insignificant terms in the model could actually be significant in the absence of other terms due to confounding. So, we carried out a deviance analysis with predictors sequentially included in the model. The analysis of deviance in Table 2 reveals that age, location, and BSc degree are statistically significant at 0.10% level of significance; gender and being employed are statistically significant at 1.0% level of significance; number of social media accounts is statistically significant at 5.0% level of significance; MSc degree, African traditional worshipping and Buddhism are statistically significant at 10.0% level of significance; other predictors are not statistically significant at any level of significance. Hence, age, location, BSc degree, gender, employment, and the number of social media accounts are the most significant predictors for the level of awareness.

From the odds in Table 1, we can observe that for each one-unit increase in age the odds of full awareness is likely to decrease by $9.9\times 10^{-1}$, supporting previous research by Willems and Mano [20]; being a male is likely to increase the odds of full awareness by 1.7644, not supporting previous research by Modi et al. [15]; living in a city will likely increase the odds of full awareness by 1.7121; having a BSc degree is likely to increase the odds of full awareness by 2.1268; being employed is likely to increase the odds of full awareness by 3.4008, suggesting that adequate sensitization about this pandemic was carried out in various government ministries and agencies, industries, and private cooperate organizations during the outbreak. Each one-unit increase in the number of social media accounts is likely to increase the odds of full awareness by 1.0908. Furthermore, people whose occupation is business were not expected to have full awareness, suggesting that there could be a high risk of COVID-19 contraction within different business facilities and environments since their owners/operators were less likely to ensure full compliance of the COVID-19 control and prevention protocols.

**Table 1:** Fitted logistic regression model

$\hbox {Variables}^\text {[VIF]}$	Coefficients(log odds)	Odds	Std. errors	$95\%$ CI(log odds)	$95\%$ CI(Odds)	*z* value	$\Pr (>|z|)$^a^
$\hbox {Age}^{[1.221533]}$	${\widehat{\beta }}_1:-2.3\times 10^{-3}$	$9.9\times 10^{-1}$	$1.0\times 10^{-2}$	($-$0.0223,0.0176)	(0.9780,1.0178)	− 0.229	0.81856
$\hbox {Gender}^{[1.140526]}$	${\widehat{\beta }}_2:5.6\times 10^{-1}$	1.7644	$2.5\times 10^{-1}$	(0.0627,1.0729)	(1.0647,2.9238)	2.204	$0.02756^{\star }$
$\hbox {Location}^{[1.124206]}$	${\widehat{\beta }}_3:5.3\times 10^{-1}$	1.7121	$2.6\times 10^{-1}$	(0.0183,1.0571)	(1.0185,2.8780)	2.029	$0.04250^{\star }$
No. of. Social.$\hbox {Media}^{[1.217030]}$	${\widehat{\beta }}_4:8.6\times 10^{-2}$	1.0908	$7.4\times 10^{-2}$	($-$0.0586,0.2325)	(0.9430,1.2617)	1.170	0.24187
Education::$\hbox {MSc}^{[1.431537]}$	${\widehat{\beta }}_5:6.6\times 10^{-1}$	1.9496	$4.0\times 10^{-1}$	($-$0.1356,1.4708)	(0.8732,4.3528)	1.629	0.10327
Education::$\hbox {PhD}^{[1.000000]}$	${\widehat{\beta }}_6:1.3\times 10^{1}$	7.0786	$6.4\times 10^{2}$	($-$1254.0620,1281.0020)	(0.0000,$\infty$)	0.021	0.98339
Education::$\hbox {BSc}^{[1.668772]}$	${\widehat{\beta }}_7:7.5\times 10^{-1}$	2.1268	$3.1\times 10^{-1}$	(0.1345,1.3747)	(1.1439,3.9541)	2.385	$0.01709^{\star }$
Education::$\hbox {Secondary}^{[1.541658]}$	${\widehat{\beta }}_8:8.1\times 10^{-4}$	1.0008	$3.4\times 10^{-1}$	($-$0.6744,0.6760)	(0.5095,1.9661)	0.002	0.99812
Education::$\hbox {None}^{[1.047973]}$	${\widehat{\beta }}_9:-9.4\times 10^{-1}$	$3.8\times 10^{-1}$	1.460	($-$3.8091,1.9141)	(0.0222,6.7808)	$-$0.649	0.51647
Education::$\hbox {Primary}^{[1.338356]}$	${\widehat{\beta }}_{10}:-4.9\times 10^{-1}$	$6.0\times 10^{-1}$	$6.4\times 10^{-1}$	($-$1.7601,0.7635)	(0.1720,2.1459)	$-$0.774	0.43887
Occupation::$\hbox {Employed}^{[1.098503]}$	${\widehat{\beta }}_{11}:1.224$	3.4008	$4.6\times 10^{-1}$	(0.3124,2.1356)	(1.3667,8.4621)	2.633	$0.00847^{\star \star }$
Occupation::$\hbox {Professional}^{[1.093742]}$	${\widehat{\beta }}_{12}:5.3\times 10^{-1}$	1.7105	$4.0\times 10^{-1}$	($-$0.2486,1.3222)	(0.7799,3.7516)	1.340	0.18031
Religion::$\hbox {Islam}^{[1.047534]}$	${\widehat{\beta }}_{12}:6.9\times 10^{-1}$	2.0095	$5.1\times 10^{-1}$	($-$0.3189,1.7147)	(0.7269,5.5553)	1.345	0.17858
Religion::$\hbox {Traditional}^{[1.100935]}$	${\widehat{\beta }}_{14}:-1.4110$	$2.4\times 10^{-1}$	$7.7\times 10^{-1}$	($-$2.9343,0.1123)	(0.0532,1.1189)	$-$1.816	$0.06937^{\mathbf {\cdot }}$
Religion::$\hbox {Buddhism}^{[1.027394]}$	${\widehat{\beta }}_{15}:-1.7290$	$1.7\times 10^{-1}$	$9.5\times 10^{-1}$	($-$3.6008,0.1428)	(0.0273,1.1535)	$-$1.811	$0.07017^{\mathbf {\cdot }}$
Religion::$\hbox {Hinduism}^{[1.081103]}$	${\widehat{\beta }}_{16}:-6.2\times 10^{-1}$	$5.3\times 10^{-1}$	1.565	($-$3.6972,2.4376)	(0.0248,11.4455)	$-$0.402	0.68739

^a^Significance level: $$1.0\%^{\star \star }$$, $$5.0\%^\star$$, $$10.0\%^\cdot$$

**Table 2:** Analysis of deviance for the logistic regression model fitted with predictors added sequentially

Terms	Degree of freedom	Deviance	Residual degree of freedom	Residual deviance	$\Pr \left( >\chi ^2\right)$^a^
−			537	744.44	
Age	1	228.537	536	515.90	${<2.2\times 10^{-16}}^{\star \star \star }$
Gender	1	8.452	535	507.45	$0.0036^{\star \star }$
Location	1	20.423	534	487.03	${6.2\times 10^{-6}}^{\star \star \star }$
No. of. Social.Media	1	6.075	533	480.95	$0.0137^{\star }$
Education::MSc	1	2.740	532	478.21	$0.0979 ^{\mathbf {\cdot }}$
Education::PhD	1	1.478	531	476.74	0.2242
Education::BSc	1	15.239	530	461.50	${9.4\times 10^{-5}}^{\star \star \star }$
Education::Secondary	1	0.049	529	461.45	0.8242
Education::None	1	0.341	528	461.11	0.5594
Education::Primary	1	1.307	527	459.80	0.2530
Occupation::Employed–	1	8.041	526	451.76	$0.0046^{\star \star }$
Occupation::Professional	1	1.833	525	449.93	0.1758
Religion::Islam	1	2.627	524	447.30	0.1050
Religion::Traditional	1	3.043	523	444.26	$0.0811^{\mathbf {\cdot }}$
Religion::Buddhism	1	3.292	522	440.96	$0.0696^{\mathbf {\cdot }}$
Religion::Hinduism	1	0.160	521	440.80	0.6896

^a^Significance level: $$0.1\%^{\star \star \star }$$, $$1.0\%^{\star \star }$$, $$5.0\%^\star$$, $$10.0\%^{\mathbf {\cdot }}$$

However, this study suggests that extensive sensitization was needed at the onset of this pandemic in Africa, particularly among different trade unions, to improve members’ awareness of the recommended practices for controlling and preventing the spread of COVID-19. Africa is arguably the most religious continent in the world (see Huffpost [13]). As such, huge progress could have been made on the prevention and control of COVID-19 if religious leaders were encouraged to take the responsibility of educating and sensitizing their members on the prevention and control routines for the COVID-19 pandemic whilst the development, approval by the appropriate regulatory agencies and the distribution of the vaccine were underway.

## Limitations

This study has limitations. First, it employed a convenience sampling technique with clear drawbacks. Second, it only involved social media users in some African countries. Given the poverty level in Africa, not everyone can afford the luxury of the internet on mobile devices to participate in this study. Thirdly, given that the majority of the participants are from Nigeria, the findings cannot be generalized to the whole of Africa. Lastly, this study involved multilingual participants, most of whom are citizens of some non-Anglophone countries. The majority could not participate because, the research instrument was only in the English language. Based on these limitations, future studies should consider:

1. Using a probability sampling technique;
2. Allowing participants from other groups instead of just social media users;
3. Using a study design that can boost the participation of eligible individuals in all the African countries;
4. The use of different language translations for the research instrument to encourage extensive participation.

All these improvements would ultimately result in larger sample sizes and facilitate the study’s findings’ statistical generalizability for the whole of Africa.

## Supplementary Information

**Additional file 1.** Survey.

## Data Availability

The survey questionnaire, data and the $\textsf {R}$ code used for the analysis can be obtained from the authors.
