# Supplementary material for: Socio-economic and demographic impacts on the full awareness of the methods for controlling/preventing the spread of COVID-19 among social media users in some African countries at the onset of the pandemic
Source: BMC Res Notes. 2021 Aug 27;14:331. doi: 10.1186/s13104-021-05736-z (PMC8391008; doi:10.1186/s13104-021-05736-z)
Supplement: Supplementary file 1 — Additional file 1. Survey. [file 13104_2021_5736_MOESM1_ESM.pdf]

Coronavirus Disease (COVID-19): AFRICAN INSTITUTE FOR MATHEMATICAL SCIENCES (AIMS), MBOUR- SENEGAL.

May we appeal to you to support our research by completing this questionnaire on COVID-19. The aim of the research is to study peoples awareness level on the methods of preventing and controlling the spread of COVID-19 disease in Africa. We assure you that the questions are non-sensitive, completely anonymized and does not induce any undue psychological stress or anxiety. Please kindly tick below to confirm your consent to participate in this survey before.

* Required

Are you happy to participate in this survey? *

Yes
No

Thank you for agreeing to complete this questionnaire. The whole question can take approximately 8 minutes to answer. We are aware of the global data protection act of 2018 therefore, we assure you that the information that you are about to provide herein will only be used for the exclusive purpose of academic research. Are you 12 years old or above? *

Yes
No

Are you currently in Africa? *

Yes
No

Which Country in Africa are you in? *

Choose

How old are you? *

Choose

How many social media platforms/account E.g.: Facebook, WhatsApp, Twitter, Instagram, Telegram, etc. do you operate? *

Choose

What is your gender? *

Female

Male

What is your highest level of education? *

No formal education
Primary
Secondary
Diploma
BSc
MSc
PhD

What is your religious affiliation? *

Buddhism
Hinduism
Islam
Christian
Traditional worshipper
None

What is your occupation status? *

Business
Employed
Professional
None

Gender *

Female
Male

Are you aware of all the following recommended practices for the prevention and control of the spread of COVID-19: 1) Quarantine (Self-isolation), 2) Isolation, 3) Staying at home unless it is necessary to go to work, 4) Avoiding all non-essential travels, 5) Avoiding group gatherings, 6) Limiting contact with people at higher risk such as older adults and those in poor health, 7) Avoiding common greetings such as handshakes, 8) Going outside to exercise but, close to the home, 9) Keeping a distance of approximately 2 meters from others outside your home, 10) Regular hand washing with soap and water for at least 20 seconds especially after using the washroom and when preparing food or using the alcohol -based hand sanitizer in the absence of soap and water, 11) Coughing or sneezing into a tissue or bent elbow, 12) Disposing of any tissues as soon as possible in a lined waste basket and washing hands afterwards, 13) Avoiding the touching of the eyes, nose or mouth with unwashed hands, 14) regular cleaning of tops and surfaces with the appropriate disinfectant and wearing of masks or face covering. *

Yes
No
